# First District Dental Society of the State of New York

**Published:** 1886-05

**Authors:** 


### FIRST DISTRICT DENTAL SOCIETY OF THE STATE OF NEW YORK.

REPORTED EXPRESSLY FOR THE INDEPENDENT PRACTITIONER.

This society held its regular monthly meeting at the rooms of the S. S. White Dental Manufacturing Co., on the evening of April 6th, with the president, Dr. Wm. Carr, in the chair. The report from the afternoon clinic was as follows:

The attendance was about ninety. Dr. G. H. Dickey, of Brooklyn, presented a young lady about nine years of age, with very badly atrophied teeth. The advice given was to grind off the cutting edges and labial surfaces of all the incisor teeth, and thoroughly polish them.

Dr. C. H. Moseley, of Brooklyn, exhibited his new anæsthetic, called Soporative, which, it is claimed, only requires from seven to twenty-two inhalations to produce perfect anæsthesia, lasting from thirty to sixty seconds. At the clinic it was given to one patient for whom three teeth were extracted, and it was also administered to several dentists as an experiment. Dr. Moseley has used the anæsthetic for over five years, and has administered it to more than three thousand patients.*

Dr. Hamilton Barnes demonstrated the lining of rubber plates with heavy gold foil, especially prepared for the purpose.

Dr. Fr. Abbott, of Brooklyn, presented a patient for whom he had inserted a very large filling, involving the mesial and grinding surface of a right upper first molar tooth, filled in twenty-three minutes, with the new form of gold which was introduced by Dr. F. A. Brauneis, of New York City, a few months ago. The margins of the filling appeared to be good, although it has been shown by experiments that the adaptation of this gold to the walls of the cavity is imperfect.

Dr. Crowell, of New York, exhibited some pieces of 18-karat gold, as well as platinum, upon which some of his new body and gum enamel had been flowed. Both require only a dull red heat for complete fusion, and are claimed to be much stronger than the ordinary continuous-gum materials.

Dr. Starr, of the S. S. White Dental Manufacturing Co., exhibited a small box for holding pluggers and excavators, with a sliding

* We do not know of what Dr. Moseley's anæsthetic consists, but it is therapeutically certain that no new agent has lately been discovered. The drugs which produce anæsthesia are all well known, and their character has long been established.—EDITOR.

lid, and a box containing three glass jars for holding polishing material, such as pumice, chalk, etc., for office use.

Dr. Verplank, of Albany, showed a new rubber dam holder, designed to depress the rubber in the lingual portion of the mouth.

Mr. Fred Feltner exhibited the carbolized preparations recommended by A. Witzel for pulp treatment. These preparations, which are manufactured by S. Brechtel & Co., of Nurnberg, Germany, are put up in six half-ounce bottles, of which the first contains a carbolized solution of mastics employed upon cotton for temporary fillings ; the second a carbolized pulp varnish, to be applied to exposed pulps previous to capping, and the third a carbolized solution of chloride of zinc, with iodine and morphine, employed for obtunding sensitive dentine, as well as in cases in which an escharotic agent is required. It is claimed that this preparation is a better escharotic than a strong solution of chloride of zinc, and that it acts without producing pain. The fourth bottle contains a carbolized solution of arsenious acid with morphine, recommended as a mild escharotic, the fifth a paste composed of arsenious acid and morphine, for devitalizing pulps, and the sixth a carbolized cement for capping exposed pulps and filling root canals. These preparations are extensively used in Germany.

Dr. Wm. H. Mitchell, of Bergen Point, N. J., presented the models of a case of irregularity, and several sizes of a new impression cup, designed for crowns and small bridge work.

Dr. E. P. Brown, of Flushing, L. I., exhibited samples of a new lot of his depressed rubber dam, which was exceedingly tough.

Dr. C. F. W. Bödecker, of New York, exhibited some teeth and matrices sent to the clinic by Dr. Wm. Herbst, of Bremen. The filled teeth beautifully illustrated the combined use of gold and amalgam for teeth with thin labial walls, where amalgam, when used alone, would discolor the teeth. The most important of these preparations were: A lower molar with a very large cavity in its grinding surface, which was lined with a thin layer of gold. An upper molar with a large cavity in the grinding surface, lined with gold and partially filled with tin. An upper bicuspid with a very large cavity, involving the mesial and grinding surfaces, lined with a thin layer of gold. Around this tooth there was a band matrix made of German silver. An upper lateral incisor with a cavity in the mesial and lingual surfaces, lined with gold. One lower and two upper central incisors, lined with gold and filled with Herbst's amalgam. An upper bicuspid with a very large gold filling, involving about one-half of the entire crown of the tooth. The filling was left unfinished, and as the German silver matrix in which the filling was made was in position, but could be moved, it was noticeable that nothing had been done to the gold extending over the edges of the cavity after the removal of the matrix, and yet not the slightest defect was visible. There was also a medallion of a cameo, representing a head, which was made of a very thin layer of gold and Herbst's amalgam. This piece demonstrated that by the Herbst method gold can be burnished into the finest lines and irregularities of cavities, and that a thin layer of gold is not affected by amalgam, if a thin coating of varnish is applied, and that, consequently, amalgam, under such circumstances, will not discolor thin walls of teeth. The procedure of the method, which is original with Herbst, is as follows: After the cavity has been prepared and dried, a piece of No. 30 to 60 very soft gold foil (for this purpose Wolrab's is preferable), in size a little larger than the cavity, is pressed into it with a piece of cotton large enough to occupy the entire cavity. Then with a smooth-faced burnisher, rotated by the engine, the cotton is pressed into every corner. When the cotton is removed if the gold does not lie firmly against the wall in all parts of the cavity, a piece of No. 4 tin foil rolled up may be employed in the same manner as the cotton. If the gold foil has been perforated during the introduction, it can be repaired by small pieces of No. 30 to 60 gold foil, previously annealed, when finally, another large layer of the same foil is applied in the same manner. The gold in the cavity is then to be covered with a thin coat of suitable varnish, such as balsam of fir, and when dry the amalgam is introduced. When tin has been used for the compression of the gold it may, if the size and shape of the cavity will admit, be left in situation over the gold, but in this case a thin coating of varnish has to be applied to the tin.

Dr. Morey exhibited a case of fusion of two germs, in the situation of the right upper central incisor, in the mouth of a boy about seven years of age.

Dr. Atkinson, in the absence of Dr. B°decker, announced that Dr. Wm. Herbst, of Bremen, will give a clinic of three or four days' duration in the month of July.

